# An *in silico* prediction tool for the expansion culture of human skeletal muscle myoblasts

**DOI:** 10.1098/rsos.160500

**Published:** 2016-10-26

**Authors:** Yuki Kagawa, Masahiro Kino-oka

**Affiliations:** Department of Biotechnology, Graduate School of Engineering, Osaka University, 2-1 Yamada-oka, Suita, Osaka 565-0871, Japan

**Keywords:** computational modelling, autologous skeletal myoblast, cellular automaton, kinetic model, cell migration

## Abstract

Regenerative therapy using autologous skeletal myoblasts requires a large number of cells to be prepared for high-level secretion of cytokines and chemokines to induce good regeneration of damaged regions. However, myoblast expansion culture is hindered by a reduction in growth rate owing to cellular quiescence and differentiation, therefore optimization is required. We have developed a kinetic computational model describing skeletal myoblast proliferation and differentiation, which can be used as a prediction tool for the expansion process. In the model, myoblasts migrate, divide, quiesce and differentiate as observed during *in vitro* culture. We assumed cell differentiation initiates following cell–cell attachment for a defined time period. The model parameter values were estimated by fitting to several predetermined experimental datasets. Using an additional experimental dataset, we confirmed validity of the developed model. We then executed simulations using the developed model under several culture conditions and quantitatively predicted that non-uniform cell seeding had adverse effects on the expansion culture, mainly by reducing the existing ratio of proliferative cells. The proposed model is expected to be useful for predicting myoblast behaviours and in designing efficient expansion culture conditions for these cells.

## 1. Introduction

Cell culture is one of the most basic but essential processes for cell-based regenerative therapies. Recently, many studies on the culture-expansion process of stem cells, including tissue-specific stem cells, embryonic stem cells and induced pluripotent stem cells, have been reported. *In vitro* culture of skeletal muscle-derived myoblasts, the progeny of quiescent mononucleated muscle precursor cells (satellite cells), has also been extensively investigated. Such studies have subsequently led to clinical success of myocardial regeneration therapy following autologous skeletal myoblast transplantation [1–4]. In addition, for the future treatment of muscular dystrophies, allo- and autotransplantations of myoblasts have been investigated [5–8].

In myocardial regenerative therapy, transplanted myoblasts are thought to secrete cytokines and chemokines which induce angiogenesis, have anti-fibrosis and anti-apoptosis effects, and recruit stem cells into the damaged regions [9–11]. Consequently, large numbers (greater than 10^8^) of myoblasts are necessary for successful cell therapy. In the case of autologous myoblasts, this requires significant cell expansion from muscle biopsy samples. To achieve a stable supply of cell-based products for regenerative therapy applications, developing a technology for the prediction of expansion cultures using autologous cells is expected. As a first step, understanding cell behaviours during the expansion process is required.

Myoblast differentiation is considered to have a dominant effect on the expansion process, because the cells lose their proliferative potential. The differentiation process, referred to as skeletal myogenesis, is considered to occur via signals initiated through cell–cell adhesions [12]. Myoblasts are then fused to each other and known lose their adhesion ability to the underlying substrate during the formation of myotubes [13]. This property of non-adherence to the culture surface has a significant effect on cell expansion in repeated subcultures. Therefore, to achieve an effective expansion culture of skeletal myoblasts, strategies for the prevention of spontaneous cell differentiation and for maintaining an undifferentiated state are required.

During *in vitro* culture of mouse myoblasts, basic fibroblast growth factor (bFGF) is known to repress their differentiation [14]. Human muscle-derived stem cells are reported to increase their rate of proliferation following addition of platelet-derived growth factor-BB combined with epidermal growth factor (EGF) and bFGF [15]. The growth rates of human myoblasts are also reported to increase in the presence of transforming growth factor-β or lysophosphatidic acid combined with bFGF [16]. Therefore, several molecules, in particular, growth factors, can enhance proliferation and repress differentiation of myoblasts *in vitro*.

In a previous study by our group, we found that disjunction time after cleavage furrow formation during cell division of human skeletal muscle myoblasts (HSMMs) was decreased with increased migration rate [17]. Based on this observation, it was assumed that the mean duration time of cell–cell adhesion was also decreased, and thus the frequency of the process towards myotube formation was decreased with increased migration rate. Using laminin-coated culture surfaces, with and without EGF supplementation, myoblast migration rate is enhanced and the frequency of cell–cell contact reduced. Therefore, myoblast expansion was enhanced by inhibiting their differentiation [17]. In addition, when HSMMs are seeded at high cell density, many seeded cells are observed to become quiescent owing to contact inhibition, similar to other anchorage-dependent cells, thus avoiding differentiation [18].

Taken together, previous studies indicate that when human myoblasts are cultured *in vitro*, these cells are composed of three subpopulations of cells, comprising those in proliferative, quiescent and post-mitotic states. In post-mitotic state, cells have initiated, but not completed their differentiation process. Considering that the composition ratio between these three states varies with time, there must be optimal seeding and subculture conditions for the expansion of a given myoblast. Our research group previously acquired data for the relationship between the degree of confluence and cell attachment during subsequent subculture, the optimized conditions for seeding density and the time to subculture, all of which were proposed and verified via *in vitro* cell culture using an automated culture system [19]. However, the proposed culture conditions were only applicable to myoblasts derived from the same batch as that used in the study from which the culture conditions were derived. Therefore, these conditions were not applicable for the expansion culture of any autologous cell type. Generally, it is very difficult to predict when and where cell differentiation will occur under a given condition, because duration time of cell–cell attachment is considered to depend not only on migration rate, but also on the local cell density, which is strongly dependent on the initial cell distribution.

For predicting such complex cell culture phenomena and designing an optimized cell culture, mathematical modelling and numerical simulations are effective strategies. In several previous studies, proliferation of anchorage-dependent mammalian cells is described by stochastic models such as cellular automata [20,21]. Based on the simulation results using such stochastic models, the effect of heterogeneity within the spatial distribution of seeded cells on growth rates has been predicted [22–24].

Our research group previously proposed a two-dimensional cellular automaton model describing monolayer keratinocyte culture [25]. By fitting the model simulation results to the observed growth curves, kinetic parameters expressing the cell culture process, such as inoculated cell adhesion, exponential growth and contact inhibition, can be estimated quantitatively [26,27]. As an extension of this model, a model describing three-dimensional culture of chondrocytes embedded in collagen gel has been developed [28,29].

In this study, we have developed a novel model describing the proliferation and differentiation process observed during *in vitro* myoblast culture, by implementing cell migration and differentiation processes into our previous two-dimensional model. The developed model will be a useful tool for the prediction of expansion culture of autologous skeletal myoblasts.

## 2. Model development

### 2.1. Two-dimensional cell placement model

The model was developed by implementing the three features of migration, quiescence and cell differentiation, which are required for describing *in vitro* culture of HSMMs, into the two-dimensional cell placement model reported previously [25]. The following assumptions were made

1. A fraction of the inoculated cells (myoblasts) can attach to the culture surface. The ratio of adherent cell concentration at *t* = 24 h to the seeding density (*t* = 0 h) is defined as 0 ≤ *α* ≤ 1.
2. A cell to be attached to the surface starts cell division after a given duration of time, required for attachment and acclimation, defined here as a lag time *t*_L_.
3. During the acclimation phase, cells neither migrate nor differentiate.
4. After the first cell division, both daughter cells repeat cell division at every generation time *τ*_g_.
5. If there is no vacant space for placement of daughter cells, then cell division of the mother cell does not occur owing to contact inhibition.
6. Cells migrate on the surface with a migration rate of *V*_m_. The rate and direction of migration are changed autonomously and by the surrounding cells through intercellular interactions.
7. A proliferative cell enters quiescent state only when the cell is in the early G1 phase of the cell cycle, and there is no vacant space around the cell. This assumption is based on the observation that only post-mitotic cells in the first 3–4 h of G1 phase entered quiescence [30].
8. A quiescent cell neither divides nor differentiates.
9. A quiescent cell instantly returns to a proliferative cell when vacant space appears around the cell.
10. When a proliferative cell attaches to one of the neighbouring cells for a given critical period of time (differentiation time *t*_dif_), this cell initiates the differentiation process and makes a strong connection to the neighbouring cell.
11. The strong connection(s) made by the differentiation process never break.
12. The maximum number of strong connections made by the differentiation process is two per cell.
13. A cell that initiated the differentiation process is called a ‘post-mitotic cell’. This cell neither divides nor returns to a proliferative state.
14. Cell death and cell removal do not occur during the culture period.

To implement these assumptions, we used a two-dimensional cellular automaton consisting of an *N* × *M* two-dimensional array of squares having a finite number of possible states that interact with neighbouring squares. Periodic boundary conditions were applied to this two-dimensional calculation space. The squares change their state at every time step, based on the assigned rules. To make a model based on the cellular automaton, the following assumptions were also made.

15. The area of each square is equal to the mean area of a single myoblast, *A*_c_. Therefore, a single cell is described by a square with a side of $\sqrt{A_{c}}$, and the distance between the squares of the neighbouring cells is $\sqrt{A_{c}}$ or $\sqrt{2A_{c}}$ (between diagonally placed squares).
16. Each square positioned at (*i*, *j*) (*i* = 1, 2, … , *N*; *j* = 1, 2, … , *M*) has a state variable *ξ*(*i*, *j*) representing one of the following states: zero for vacant space, one, two and three for occupied with a proliferative cell, quiescent cell and post-mitotic cell, respectively. In a case considering obstacles, such as a wall of a culture dish into which cells cannot migrate, a negative integer (−1) was assigned.

**Figure 1.**
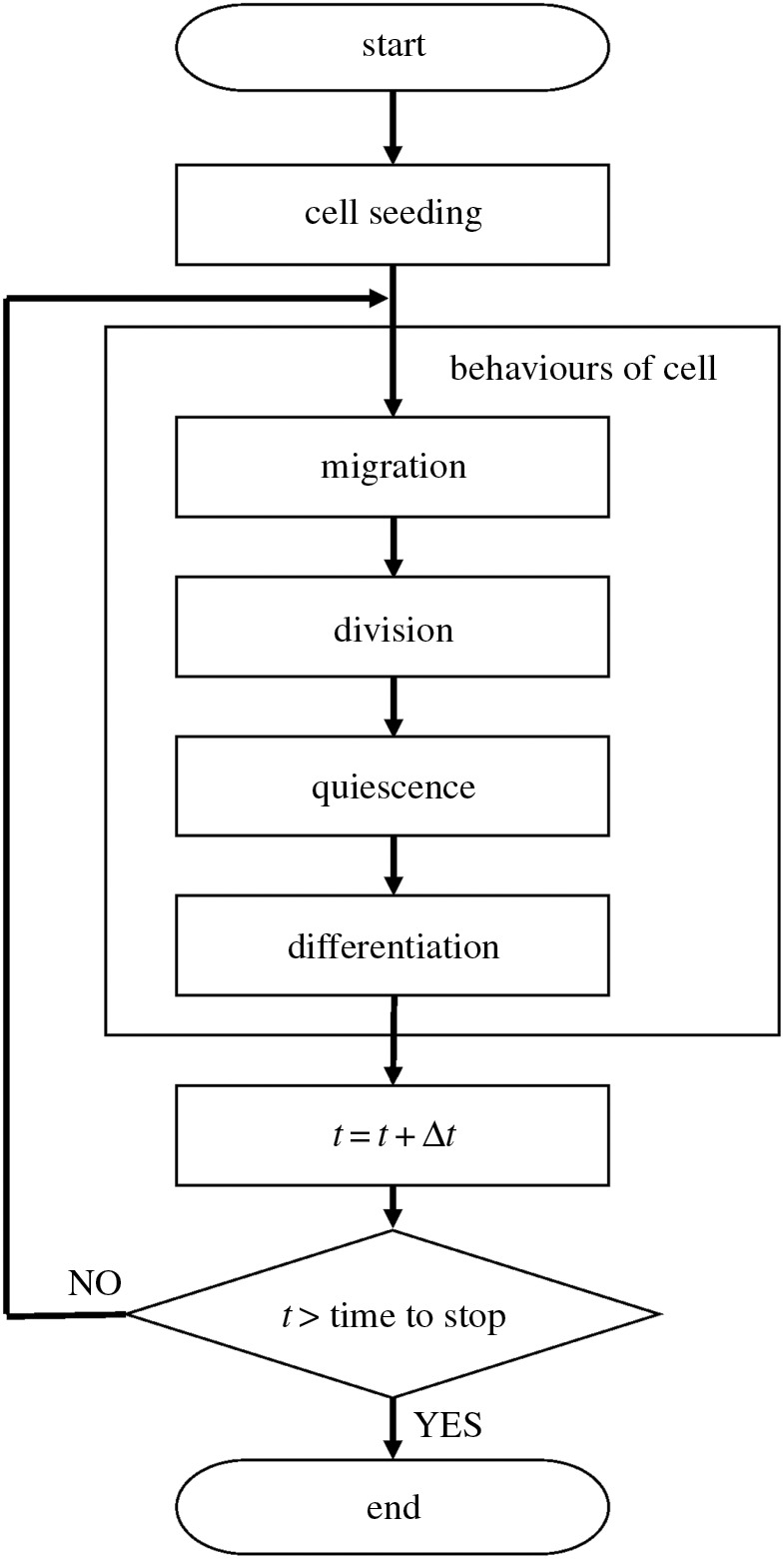
Flow of calculations for *in silico* myoblast expansion culture.

The flow of the calculation is shown in figure 1. To initialize simulation, cells were seeded onto the two-dimensional calculation space. This seeding process, comprising attachment and acclimation of part of the seeded cells onto the culture surface, can be explicitly described using adhesion time, lag time (*t*_L_) and attachment ratio (*α*), as described previously [25]. However, in this study, *α* × *X*_a,seed_ × *A*_c_ × *N* × *M* proliferative cells were put randomly onto the two-dimensional space at the time *t* = *t*_L_, where *X*_a,seed_ was a seeding cell density. After seeding, each cell executed the four cell behaviours (migration, division, quiescence and differentiation) defined by the specific rules described below. When all cells executed all four cell behaviours, *t* is increased by a time step Δ*t* of 0.1 h. The calculation was finished when *t* reached a predetermined time. In the following, the rules of each cell behaviour are described in greater detail.

### 2.2. Rules of migration

Cells can migrate in one of eight distinct directions denoted by the variables dir, which are assigned from north (dir = 0) to northwest (dir = 7) in clockwise order. For each time step, a cell moves to one of the eight nearest neighbour (NN) squares or stays within the current square. To describe the migration of cell *c* in the direction dir with the rate of *V*_m,*c*_, first the cell migrates to the NN square located in the direction dir of the current square, and second the variable *t*_m,*c*_ is updated, a waiting time for the next migration, as $t_{m,c}=l\;V_{m,c}^{-1},$ where *l* is the migration distance given by $l=\sqrt{A_{c}}$ when dir is even or $\sqrt{2A_{c}}$ when dir is odd. The waiting time *t*_m,*c*_ decreases by Δ*t* for each time step. If the waiting time is maintained (*t*_m,*c*_ > 0), then this cell does not actively migrate, but can be passively exchanged with the NN cells whose waiting times are less than zero.

When the waiting time of cell *c* becomes less than zero (*t*_m,*c*_ *≤* 0), the direction dir and the rate *V*_m,*c*_ of migration are determined by the following rules. First, the direction dir is determined stochastically based on the probability Pr_m,dir_. This probability is determined by the number of weak connections to be broken *n*_dis,dir_ as a result of displacement in the direction dir. Whenever a cell is placed next to another cell, these two neighbouring cells are considered to be weakly connected. When a cell connects with the NN cell placed at the direction dir of the former, the number of weak connections between these cells is defined as *n*_e_ when dir is even, and *n*_o_ (≤*n*_e_) when dir is odd. Therefore, if the cell moves to the even direction (dir = 0, 2, 4, 6), then the total number of broken weak connections is *n*_dis,dir_ = 3*n*_e_ (derived by subtracting the total number of weak connections after cell displacement, *n*_e_ + 4*n*_o_, from that before displacement, 4*n*_e_ + 4*n*_o_) under confluent condition (whereby all squares are occupied with cells). Under the same condition, if the cell moves in the odd direction (dir = 1, 3, 5, 7), then the total number becomes *n*_dis,dir_ = 2*n*_e_ + 3*n*_o_ instead. In this study, we assumed *n*_e_ = *n*_o_ = 1.

We defined the weight *R*_m,dir_ for accepting migration in the direction dir as

2.1 $$R_{m,\mathrm{dir}}={F_{c}}^{-n_{\mathrm{dis},\mathrm{dir}}},$$

where *F*_c_ is a dimensionless parameter representing the strength to break a single weak connection. When any strong connections are broken as a result of displacement in the direction dir, *R*_m,dir_ is forced to be assigned with zero, regardless of the value of *n*_dis,dir_. Next, by using these weight values, we calculate

2.2 $$p_{m,\mathrm{dir}}=\frac{R_{m,\mathrm{dir}}}{\sum_{d}R_{m,d}},$$

for all dir directions. Finally, we have the probability to select dir as a migration direction as

2.3 $$Pr_{m,\mathrm{dir}}=\frac{{\left(p_{m,\mathrm{dir}}\right)}_{C}{\left(p_{m,\tilde{\mathrm{dir}}}\right)}_{N}}{\sum_{d}[{\left(p_{m,d}\right)}_{C}{\left(p_{m,\tilde{d}}\right)}_{N})]},$$

where subscripts C and N of the parentheses represent the values at the current and its neighbouring square, respectively. $\tilde{\mathrm{dir}}$ and $\tilde{d}$ represent the directions opposite to dir and *d*, respectively. If the neighbouring square is vacant, then the value is set to unity ${\left(p_{m,\tilde{\mathrm{dir}}}\right)}_{N}=1$. When the cell *c* moves to the neighbouring square, its migration rate is also updated as follows.

2.4 $$V_{m,c}=V_{\max}-(V_{\max}-V_{\mathrm{conf}})\frac{5\;n_{\mathrm{dis},\mathrm{dir}}}{4(n_{\mathrm{dis},\mathrm{dir}}+1)},$$

where *V*_max_ and *V*_conf_ are the migration rates when the cell is isolated (thereby no surrounding cells exist) and when the cell is completely surrounded by other cells (confluent state), respectively. Note that equation (2.4) was determined as a decreasing function of *n*_dis,dir_, which satisfies *V*_m,*c*_ = *V*_max_ when *n*_dis,dir_ = 0, and *V*_m,*c*_ = *V*_conf_ when *n*_dis,dir_ = 4, which is the average number of broken weak connections during migration under confluent conditions.

### 2.3. Rules of division

Each cell has a waiting time for the next division *t*_d,*c*_, which is decreased by Δ*t* for each time step. When the waiting time is less than zero, and there is at least one vacant NN square, this cell can divide and update *t*_d,*c*_ as *t*_d,*c*_ = *t*_d,*c*_ + *τ*_g_, where *τ*_g_ is a generation time. In a simulation, we determined the value of *τ*_g_ for each update by giving a random value between 0.9*t*_g_ and 1.1*t*_g_ with a uniform distribution (therefore *t*_g_ is the mean generation time). If there is no vacant NN square, then this cell cannot divide (contact inhibition) and does not update the waiting time. During cell division, the mother cell puts its daughter on one of the vacant NN squares stochastically as described previously [25]. Practically, the probability that the mother cell puts the daughter on an NN square placed at the direction dir of the mother is given by

2.5 $${\mathrm{Pr}}_{d,\mathrm{dir}}=\frac{R_{d,\mathrm{dir}}}{\sum_{\mathrm{di}r^{{\;}^{\prime }}}R_{d,\mathrm{di}r^{{\;}^{\prime }}}},$$

where

2.6 $$R_{d,\mathrm{dir}}=\left\{ \begin{array}{ll} 2, & \text{when the NN square is vacant and dir is even}\\ 1, & \text{when the NN square is vacant and dir is odd}\\ 0, & \text{when the NN square is filled by the other cell}. \end{array}\right.$$

### 2.4. Rules of quiescence

If the mother or daughter cell is surrounded by other cells, and there are no vacant NN squares, at the moment when the cell is placed as a result of cell division, then this cell enters the quiescent state. If the cell is in the quiescent state, the waiting time for the next division does not decrease for each time step. Whenever a vacant square appears at the NN of a quiescent cell, this quiescent cell returns to a non-quiescent proliferative cell state at the next time step. Quiescent cells neither divide nor differentiate.

### 2.5. Rules of differentiation

If a non-quiescent proliferative cell attaches to one of the NN cells for a critical period of time (*t*_dif_), this cell initiates the differentiation process and makes a strong connection to the NN cell, only when the NN cell can make a strong connection. Note that each post-mitotic cell can make a maximum of two strong connections. The strong connections never break, and post-mitotic cells never divide.

Symbols appearing in this article are summarized in appendix.

## 3. Material and methods

### 3.1. Cell culture

Human skeletal muscle myoblasts (Lonza, Walkersville, MD) were maintained in 75 cm^2^ T-flasks (Nulgen Nunc, Rochester, NY) containing 15 ml Dulbecco's modified Eagle's medium (Sigma-Aldrich, St Louis, MO) supplemented with 10% (v/v) fetal bovine serum (Invitrogen, Grand Island, NY) at 37°C in air containing 5% (v/v) CO_2_ by sequential subculture processes.

### 3.2. Live imaging of cell migration

Viable cells were seeded at a density of 2.3 × 10^5^ cells cm^−2^, with a small number of cells (75 cells cm^−2^; 0.033%) stained with CellTracker™ Green (Invitrogen) to track migration of individual cells. After 36 h of seeding, migration of individual myoblasts was observed using a confocal laser scanning microscope (FV10i, Olympus, Tokyo, Japan) with a 10× objective lens every 10 min at nine positions for 1 h. The migration rate of each cell was determined by measuring the distance between centroids of a stained cell on the images captured at different time points as described previously [17].

## 4. Results and discussion

### 4.1. Estimation of the model parameters

To predict cell growth of specific cell types under specific environments by numerical simulation of the model, we have to estimate the model parameter values before executing the simulations.

The length of a side of the square $\sqrt{A_{c}}$ was assumed to be the square root of the averaged cell size. In the case of HSMMs, the average area occupied by a single cell was estimated to be 1.8 × 10^3^ µm^2^, calculated by the area of culture surface (632 cm^2^) multiplied by the confluence degree (0.48) divided by the cell number (1.7 × 10^7^), using previously reported data [19]. Confluence degree was defined as the ratio of the area occupied by the cells to the entire area of the culture surface. Therefore, the length was estimated to be 42 µm.

The mean generation time *t*_g_ can be calculated by ln(2) divided by the true specific growth rate. Using the reported value (0.033 h^−1^) [19], we estimated *t*_g_ as 21 h.

The following three parameters, mean migration rate of isolated cells *V*_max_, mean migration rate in confluent state *V*_conf_ and connection strength *F*_c_, are all related to the cell migration process. Based on our previous report [17], *V*_max_ was estimated to be 23 µm h^−1^. *V*_conf_ was directly measured by the experiment executed in this study, and was estimated to be 12.8 ± 7.4 µm h^−1^ (*n* = 22). Thus, in the model, we set *V*_conf_ as 13 µm h^−1^.

**Figure 2.**
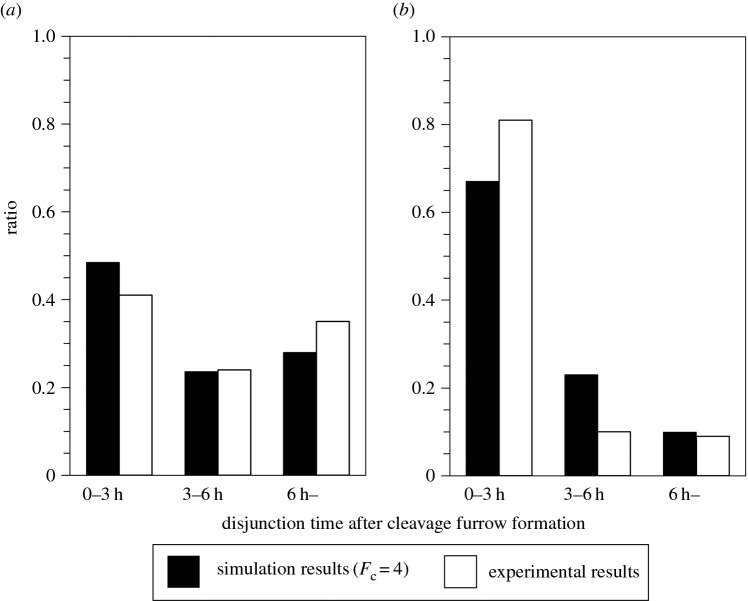
Estimation of connection strength *F*_c_ by fitting to histograms of disjunction time distribution obtained under two conditions. Histograms of disjunction times after cleavage furrow formation in each cell division obtained on plain (*a*) and laminin-coated (*b*) surfaces are shown. White bars: experimental results reproduced from the data published in our previous study [17]. Black bars: simulation results when *F*_c_ was set to 4, with which the simulation results were best fit to the experimental data. For the simulations of plain and laminin, the maximum myoblast migration rate (*V*_max_) was set to 23 and 39 µm h^−1^, respectively.

Connection strength was estimated by fitting to histograms of disjunction time after cleavage furrow formation observed during cell division [17]. Here we used histograms obtained under the following two conditions; (i) on a plain surface and (ii) on a laminin-coated surface. These histograms were reproduced from data published in our previous study [17] and are shown in figure 2*a,b* (white bars). To obtain corresponding simulation data, we executed the following simulations. Only two cells were seeded on a space of 5 × 5 squares with periodic boundary conditions, and the cells did not divide, quiesce or differentiate. For the given parameter *F*_c_, we collected much data on the duration of any contact between the two cells to create a histogram. We then searched for the histogram which had the least-square fit to the experimental results (figure 2*a,b*, black bars). For simulations on a laminin-coated surface, *V*_max_ was set to 39 µm h^−1^. From these simulations, the *F*_c_ value was determined to be 4.

**Figure 3.**
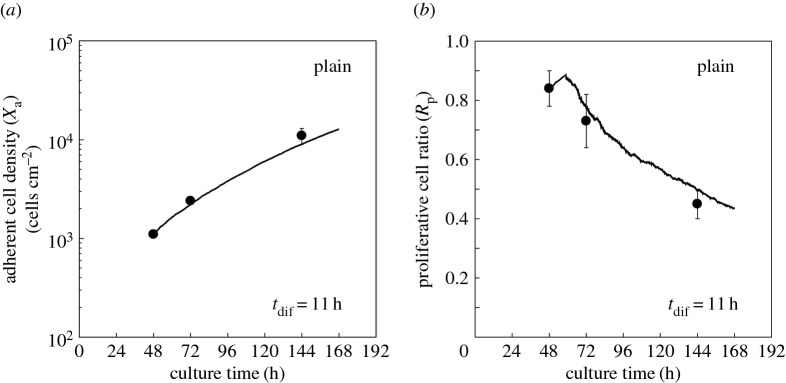
Estimation of differentiation time *t*_dif_ by fitting to time courses of adherent cell density and proliferative cell ratio obtained on a plain surface. Closed circles: experimental data of adherent cell density (*X*_a_) measured in cells cm^−2^ (*a*) and proliferative cell ratio (*R*_p_) (*b*) obtained in the previous study [19], plotted against culture time. Error bars represent standard deviations. Solid lines: simulation results when *t*_dif_ was set to 11 h, with which the simulation results were best fit to the experimental data.

The last parameter to be estimated was the differentiation time *t*_dif_. Using the estimated values of $\sqrt{A_{c}}$, *t*_g_, *V*_max_, *V*_conf_ and *F*_c_, we started numerical simulations of the model with the adherent cell density of *X*_a_ = 1.1 × 10^3^ cells cm^−2^ and proliferative cell ratio of *R*_p_ = 0.84. These data are reported from HSMM culture on a plain surface obtained at *t* = 48 h [19], and predicted the subsequent time evolutions of *X*_a_ and *R*_p_. In this simulation, it was assumed that all non-proliferative cells at *t* = 48 h were post-mitotic cells, based on the occupation ratio of cells in a culture dish at *t* = 48 h, calculated as *X*_a_ × *A*_c_ = (1.1 × 10^3^ cells cm^−2^) × (1.8 × 10^−5^ cm^2^ cell^−1^) = 0.02. With this occupation ratio, if we assume that a part of non-proliferative cells are not differentiated but in the quiescent state, these quiescent cells will return to a non-quiescent proliferative state in the next time step (i.e. after 0.1 h), and the experimental condition of *R*_p_ = 0.84 will not be able to be maintained. We searched for the parameter value of *t*_dif_, which the model predicted time evolutions of *X*_a_ and *R*_p_ which were the least-square fit to the reported values at *t* = 72 and 144 h [19] (figure 3*a,b* closed circles and solid curve). As a result, *t*_dif_ was estimated to be 11 h.

### 4.2. Validation of the model

Using the above parameter fittings, the growth curves can be simulated for a given initial condition. Here, we simulated the growth curve of myoblasts cultured on a laminin-coated surface and compared the results with experimental data reported previously [19].

When myoblasts were cultured on a laminin-coated surface, the mean migration rate was known to become faster than that on a plain surface, and was estimated to be 39 µm h^−1^ [17]. Therefore, *V*_max_ should be increased to this estimated value. All the other parameter values were assumed not to be changed using laminin-coated surfaces. In the validation, we started numerical simulations with the conditions of *X*_a_ = 1.4 × 10^3^ cells cm^−2^ and *R*_p_ = 0.86. These data are reported from HSMM culture on a laminin-coated surface obtained at *t* = 48 h [19], and predicted the subsequent time evolutions of *X*_a_ and *R*_p_.

**Figure 4.**
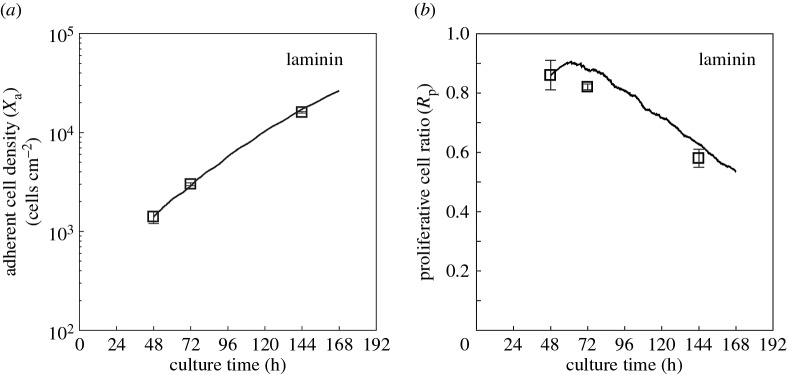
Model validation by comparing simulation results with experimental data. Open squares: experimental data of adherent cell density (*X*_a_) measured in cells cm^−2^ (*a*) and proliferative cell ratio (*R*_p_) (*b*) obtained on a laminin-coated surface [19], plotted against culture time. Error bars represent standard deviations. Solid lines: simulation results when the parameter values determined for the current culture condition were used.

As shown in figure 4*a*, the growth curve obtained in the simulation was very similar to the experimental data. Furthermore, the simulated tendency of *R*_p_ against culture time was in good agreement with that of the experimental data, although the absolute quantities were a little larger than the experimental data at all time points (figure 4*b*). As one possible cause for this discrepancy, we considered that not all attached proliferative cells could incorporate BrdU. Because the duration of incubation with BrdU was 12 h [19], whereas the generation time was estimated to be 21 h, a small number of normal proliferative cells could not incorporate BrdU if the DNA-synthesis phase was less than 9 h. Here, it should be noted that all model parameter values were determined using obtained experimental data, and the developed model was absent of adjustable parameters.

**Figure 5.**
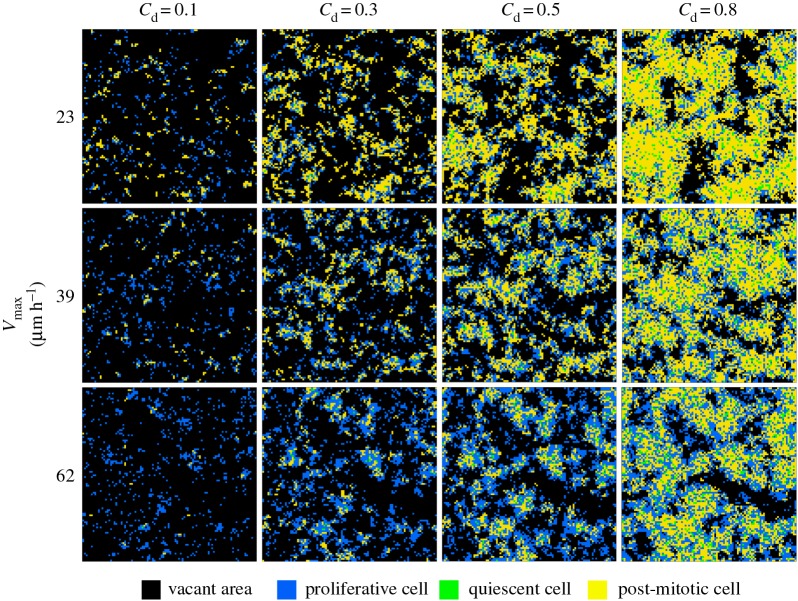
Snapshots of cell distribution at various confluence degrees. Each square of two-dimensional calculation space represents one of the following four entities; vacant area (black), proliferative cell (blue), quiescent cell (green) and post-mitotic cell (yellow).

Next, we investigated the effect of increasing the migration rate of myoblasts on the distribution of each cell type in a two-dimensional space at given confluence degrees (*C*_d_). We executed the simulations starting from the same initial condition of *X*_a_ = 1.0 × 10^3^ cells cm^−2^, *α* = 0.85 and *t*_L_ = 24 h. We compared the cell distributions at *C*_d_ = 0.1, 0.3, 0.5 and 0.8 obtained in the cases of *V*_max_ = 23 (corresponding to the cases on the plain surface), 39 (on the laminin-coated surface) and 62 µm h^−1^ (on the laminin-coated surface with EGF-supplementation to the culture medium) [17]. As shown in figure 5 (together in electronic supplementary material, S1–S3 for the above three conditions of *V*_max_ = 23, 39 and 62 µm h^−1^, respectively), the ratio of post-mitotic cells at any given *C*_d_ became smaller when the migration rate increased. This implies that the simulation of the developed model qualitatively recapitulated our previous experimental results that the existence of laminin and/or EGF led to the promotion of myoblast growth while keeping a high proliferative cell ratio by reducing the frequency of cell–cell contacts during cytokinesis, thus suppressing differentiation [17]. Taken together, for all *in silico* cultures under different conditions, the results of the simulations gave satisfactory agreements with the values obtained by the *in vitro* experiments.

### 4.3. Predictions made by the model

In several previous studies using mathematical models to determine expansion of anchorage-dependent cells, initial cell distribution was found to strongly affect the subsequent growth, and non-uniform seeding was found to slow down the increase in cell number owing to contact inhibition [22]. Simulations also predicted that this adverse effect of non-uniform seeding became severe only when cell motility was small and thus the effect can be neglected in cases using cells with high motility [23]. In the case of myoblast expansion, the effect of initial cell distribution on the subsequent growth is not obvious, because the growth rate depends on contact inhibition and on both cell migration and differentiation. In the following, by executing computer simulations of the developed model, we predicted the growth of myoblasts when the cells were seeded non-uniformly. We compared these predictions with the results obtained when the cells were seeded uniformly.

We started the simulation with a randomly given initial cell distribution on the two-dimensional calculation space. To simulate non-uniform distributions, we defined the probability density distribution function of

4.1 $$p(x)=\frac{\exp[-x/ax_{\max}]}{\left(ax_{\max})[1-\exp(-1/a)\right]}$$

in a region of *x* = [0, *x*_max_], where $x_{\max}=N\sqrt{A_{c}}$ is the length of a horizontal side of the space. This function also satisfies $\int_{0}^{x_{\max}}p(x)\;dx=1$. The uniformity of the cell distribution, *a* (>0), is defined as a variable that satisfies the following equation

4.2 $$p(ax_{\max})=\frac{1}{e}p(0).$$

This means that *p* is exponentially decreased from *p*(0) to e^−1^*p*(0) when *x* is increased from 0 to *ax*_max_. Therefore, the limit of *a* → ∞, $p(x)\to x_{\max}^{-1}$ (constant) is achieved. Using this function, the fraction of the cells seeded between *x*_a_ and *x*_a_ + Δ*x* is given by *p*(*x*_a_) Δ*x*. In the present two-dimensional cellular automaton, the probability that a square positioned at $x_{i}=(i-1/2)\sqrt{A_{c}}$ (*i* = 1, 2, … , *N*) is occupied with a cell at the time of seeding is given by $p(x_{i})\sqrt{A_{c}}$. Cell distribution along the *y*-axis (vertical coordinate) was assumed to be constant. Cells cannot migrate or divide into the regions of *x* < 0 and *x* > *x*_max_, whereas periodic boundary conditions were applied along the *y*-axis.

We considered cases in which myoblasts were seeded on a laminin-coated surface with EGF supplementation in the culture medium. Under this condition, *V*_max_ was set to 62 µm h^−1^ [17], whereas all the other parameter values were not changed. We started simulations by seeding cells on a two-dimensional calculation space composed of 240 × 40 squares with *X*_a_ = 1.0 × 10^3^ cells cm^−2^ and *R*_p_ = 1.0. For a given *a* value, we obtained a randomly generated non-uniform initial cell distribution. Practically, when *a* ≥ 1, the generated initial cell distribution was very similar to the uniform distribution. Starting from these initial cell distributions, time evolutions of spatial distribution and existing ratios of each cell type were calculated.

**Figure 6.**
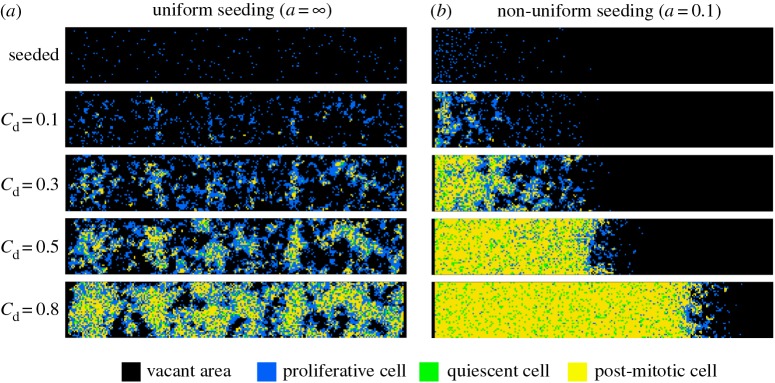
Effect of initial non-uniform seeding on subsequent myoblast growth. (*a,b*) Snapshots of cell distribution at the time points when seeding, *C*_d_ = 0.1, 0.3, 0.5 and 0.8, in the cases of uniform seeding and non-uniform seeding defined with *a* = 0.1, respectively. The colour of each square represents vacant area (black), proliferative cell (blue), quiescent cell (green) or post-mitotic cell (yellow).

Typical time evolutions of cell distributions initiated with uniform and non-uniform seeding are shown in figure 6 (together in electronic supplementary material, S4 and S5 for the conditions of uniform and non-uniform seeding, respectively). Even though we started the simulations using the same seeding density, the resultant cell distributions at given confluence degrees were very different. In the case of uniform seeding, many small growing colonies were distributed broadly in the space (figure 6*a* and electronic supplementary material, S4). On the other hand, in the case of non-uniform seeding with *a* = 0.1, one large colony grew from the left side to the right (figure 6*b* and electronic supplementary material, S5). In both conditions, many proliferative cells existed at the edge of the colonies, while post-mitotic and quiescent cells mainly existed inside the colonies. Because the area of the colony edge was small in the case of non-uniform seeding, the number of proliferative cells was relatively small.

**Figure 7.**
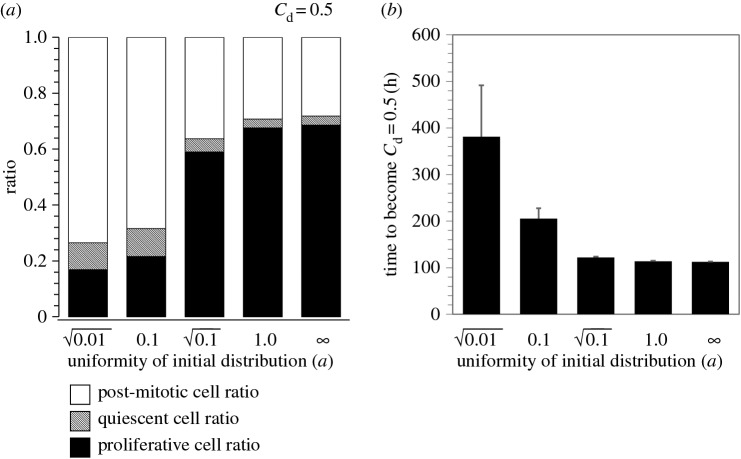
Effect of initial non-uniform seeding on the ratio of each cell type and the required time to confluence. (*a*) Existing ratios of proliferative (closed area), quiescent (striped area) and post-mitotic (open area) cells at the time when the confluence degree reaches 0.5 are arranged against the uniformity parameter of initial seeding (*a*). *a* = ∞ corresponds to uniform seeding. (*b*) Required time for the confluence degree to reach 0.5 arranged against *a*. Error bars represent standard devotions (*n* = 3; simulation runs).

Existing ratios of proliferative, quiescent and post-mitotic cells were changed drastically with uniformity of the initial cell distribution (figure 7*a*). When uniformity *a* was large enough (*a* ≥ 1), the initial distribution was almost uniform, and more than two-thirds of cells were proliferative at the time when the confluence degree was 0.5. As *a* was decreased, the existing ratio of proliferative cells was also decreased (figure 7*a*). When *a* = 0.1 (in the case shown in figure 6*b*), the ratio of proliferative cells was 0.22, less than one-third of that in the case of uniform initial seeding (0.69 when *a* = ∞). On the contrary, the existing ratios of quiescent and post-mitotic cells were increased by decreasing *a*. Non-uniform initial seeding also slowed down the increase in cell number. As shown in figure 7*b*, particularly when *a* ≤ 0.1, the required time for the confluence degree to reach 0.5 became much larger than that in the case of uniform seeding (*a* = ∞). These results imply that heterogeneous seeding should be avoided for effective expansion culture of myoblasts, because non-uniform initial seeding creates adverse effects on the culture by reducing the existing ratio of proliferative cells at a given confluence degree (figure 7*a*) and by decreasing the mean growth rate (figure 7*b*).

As shown in figure 7, these adverse effects on the expansion culture became prominent when *a* ≤ 0.1, whereas they were not prominent when $a\geq \sqrt{0.1}$. In the simulations using *a* = 0.1 and $\sqrt{0.1},$ the largest local densities of seeded cells were about 9.8 × 10^3^ cells cm^−2^ and 3.3 × 10^3^ cells cm^−2^, respectively, which were calculated by the following formula

4.3 $$\left(\mathrm{local}\;\mathrm{cell}\;\mathrm{density}\;\mathrm{at}\;x=\frac{1}{2}\sqrt{A_{c}}\;\right)=X_{a}\frac{\exp(-1/2aN)}{a[1-\exp(-1/a)]},$$

with *X*_a_ = 1.0 × 10^3^ cells cm^−2^ and *N* = 240. Therefore, when the cell density exceeds the critical value (likely to be between 3.3 × 10^3^ and 9.8 × 10^3^ cells cm^−2^) even locally, the above adverse effects can become prominent. Therefore, by considering heterogeneity in cell density that can occur at the time of seeding [31], the seeding density of *X*_a_ = 1.0 × 10^3^ cells cm^−2^ designed for the automated myoblast culture system [19] was thought to be reasonable.

It should be noted that the results shown in figures 6 and 7 were derived under the condition of *V*_max_ = 62 µm h^−1^, in which myoblasts were seeded on a laminin-coated surface with EGF supplementation to the culture medium [17]. When myoblasts are seeded on a plain surface or a laminin-coated surface with no EGF supplementation, they migrate slowly (*V*_max_ = 23 µm h^−1^ and 39 µm h^−1^, respectively). In these cases, it is considered that the proliferative cells were likely to attach to one of their NN cells for a relatively longer period of time and differentiation occurred more frequently. Accordingly, the critical local cell density is thought to become smaller.

## 5. Conclusion

To optimize culture conditions for effective expansion of skeletal myoblasts, it is desirable to be able to predict the expansion process, particularly when autologous cells are used, because cell growth is strongly dependent on the growth properties of a given sample. Here we developed a computational model describing the expansion process of myoblasts based on our previous two-dimensional cell placement model. In this study, we assumed that cells initiated differentiation when they attached to each other for a defined period of time. Although the assumption of differentiation was simple, the developed model satisfactorily described the available experimental data quantitatively. This implies that the model can be used to predict cell number and proliferative cell ratio during expansion culture using a given sample of myoblasts. With computer simulations using the developed model, we also predicted the effect of non-uniform seeding on subsequent cell expansion and found this slowed expansion down. Predictions made by these *in silico* simulations will be useful for designing an efficient culture system for expansion culture of autologous myoblasts in the future.

## Data accessibility

All data are presented in the main text.

## Appendix: glossary

*A*_c_: mean area of a single myoblast
*C*_d_: confluence degree (ratio of the area occupied by cells to the entire area of culture surface)
*F*_c_: connection strength
*N*, *M*: number of squares along horizontal and vertical axes, respectively
Pr_d,dir_: probability that the mother cell puts the daughter cell on an NN square placed in the direction dir of the mother; defined by equation (2.5)
Pr_m*,*dir_: probability that the cell migrates in the direction dir
*R*_d*,*dir_: weight for putting the daughter cell on an NN square placed at the direction dir; defined by equation (2.6)
*R*_m*,*dir_: weight for accepting the migration in the direction dir; defined by equation (2.1)
*R*_p_: proliferative cell ratio
*V*_conf_: migration rate when the cell is completely surrounded by other cells (confluent state)
*V*_m_: migration rate of a myoblast (cell)
*V*_m,*c*_: migration rate of cell *c*
*V*_max_: migration rate when the cell is isolated (no surrounding cells exist)
*X*_a_: adherent cell density
*X*_a,seed_: seeding cell density
*a*: uniformity of cell distribution; parameter appears in equation (4.1)
dir: variable denoting one of the eight discrete directions
$\tilde{\mathrm{dir}}$: the direction opposite to dir
*l*: migration distance
*n*_dis,dir_: number of weak connections to be broken as a result of displacement to the direction dir
*n*_e_: number of weak connections between a cell and the cell placed at the direction dir = 0,2,4,6
*n*_o_: number of weak connections between a cell and the cell placed at the direction dir = 1,3,5,7
*p*(*x*): probability density distribution function defined by equation (4.1)
*p*_m*,*dir_: normalized weight for accepting migration in the direction dir; defined by equation (2.2)
*t*: culture time
*t*_d*,c*_: waiting time for the next division of cell *c*
*t*_dif_: differentiation time
*t*_g_: mean generation time
*t*_L_: lag time
*t*_m,*c*_: waiting time for the next migration of cell *c*
*x*_max_: length of a horizontal side of the space; parameter appears in equation (4.1)
Δ*t*: time step
*α*: the ratio of adherent cell concentration at *t* = 24 h to the seeding density (*t* = 0 h)
*ξ*(*i*, *j*): state variable of the square positioned at (*i*, *j*)
*τ*_g_: generation time; randomly assigned value between 0.9*t*_g_ and 1.1*t*_g_
